# Clinical and experimental treatment of primary humoral immunodeficiencies

**DOI:** 10.1093/cei/uxae008

**Published:** 2024-02-02

**Authors:** Anna Szaflarska, Marzena Lenart, Magdalena Rutkowska-Zapała, Maciej Siedlar

**Affiliations:** Department of Clinical Immunology, Institute of Paediatrics, Jagiellonian University Medical College, Wielicka 265, Cracow, Poland; Deparment of Clinical Immunology, University Children’s Hospital, Wielicka 265, Cracow, Poland; Department of Clinical Immunology, Institute of Paediatrics, Jagiellonian University Medical College, Wielicka 265, Cracow, Poland; Deparment of Clinical Immunology, University Children’s Hospital, Wielicka 265, Cracow, Poland; Department of Clinical Immunology, Institute of Paediatrics, Jagiellonian University Medical College, Wielicka 265, Cracow, Poland; Deparment of Clinical Immunology, University Children’s Hospital, Wielicka 265, Cracow, Poland; Department of Clinical Immunology, Institute of Paediatrics, Jagiellonian University Medical College, Wielicka 265, Cracow, Poland; Deparment of Clinical Immunology, University Children’s Hospital, Wielicka 265, Cracow, Poland

**Keywords:** common variable immunodeficiency, transient hypogammaglobulinemia of infancy, selective IgA deficiency, primary humoral immunodeficiency, immunodeficiency treatment

## Abstract

Selective IgA deficiency (sIgAD), common variable immunodeficiency (CVID), and transient hypogammaglobulinemia of infancy (THI) are the most frequent forms of primary antibody deficiencies. Difficulties in initial diagnosis, especially in the early childhood, the familiar occurrence of these diseases, as well as the possibility of progression to each other suggest common cellular and molecular patomechanism and a similar genetic background. In this review, we discuss both similarities and differences of these three humoral immunodeficiencies, focusing on current and novel therapeutic approaches. We summarize immunoglobulin substitution, antibiotic prophylaxis, treatment of autoimmune diseases, and other common complications, i.e. cytopenias, gastrointestinal complications, and granulomatous disease. We discuss novel therapeutic approaches such as allogenic stem cell transplantation and therapies targeting-specific proteins, dependent on the patient’s genetic defect. The diversity of possible therapeutics models results from a great heterogeneity of the disease variants, implying the need of personalized medicine approach as a future of primary humoral immunodeficiencies treatment.

## Introduction

**Figure 1.**
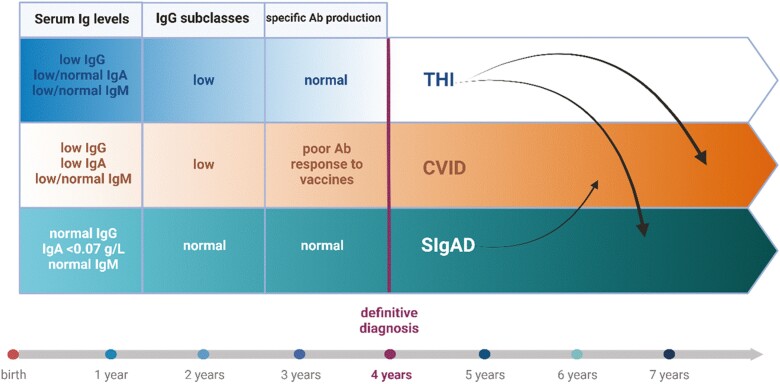
Comparison of the most common forms of primary antibody deficiencies: THI, CVID, and sIgAD, including main characteristic features and the possibility of progression to each other (arrows). Created with BioRender.com

Selective IgA deficiency (sIgAD) and common variable immunodeficiency (CVID) are the most frequent forms of primary antibody deficiencies. The occurrence of both diseases in affected families, and the possible progression from sIgAD to CVID (occasionally CVID to sIgAD) suggests a similar genetic background of these two diseases, at least in some cases. Familial inheritance of either sIgAD or CVID occurs in about 20% [1]. Common feature of both sIgAD and CVID, which might be also their first clinical manifestation, is the occurrence of autoimmune condition. In early age, CVID must be distinguished from another form of humoral immunodeficiency—transient hypogammaglobulinemia of infancy (THI). THI, in majority, recovers spontaneously around 4 years of age, whereas CVID requires immunoglobulin replacement usually throughout life. Only occasionally, THI may persist longer, even for several years, and in such case it may transform to CVID-like disease with severe infections, low IgG levels, and functional antibody defect. THI may also convert to sIgAD or to unclassified hypogammaglobulinemia (UCH) with only slightly decreased or borderline IgG levels. Thus, although sIgAD, CVID, and THI seem distant, they have much in common (Fig. 1). Here, we discuss both similarities and differences of these three humoral immunodeficiencies, focusing on current and novel therapeutic approaches.

### Selective IgA deficiency

SIgAD defines a disorder with serum IgA levels < 0.07 g/L with normal IgM and IgG levels in individuals over 4 years of age and after exclusion of the other causes of IgA deficiency [2]. In most cases, this deficiency is clinically asymptomatic [1]. In symptomatic SIgAD patients, we observe recurrent infections of upper or, less often, lower respiratory tract and gastrointestinal tract disorders, including *Giardia intestinalis* infections, absorption disorders, lactose intolerance, coeliac disease. Some patients with sIgAD reveal allergic disorders: bronchial asthma, atopic dermatitis, allergic rhinitis, allergic conjunctivitis, urticaria, food, and drug allergies [3, 4]. In sIgAD, the frequency of autoimmune diseases is higher than in general population. Systemic lupus erythematosus (SLE) and juvenile or adult-onset rheumatoid arthritis are the most common, but thyroiditis, vitiligo, autoimmune hemolytic anemia, Graves disease, diabetes type 1 have also been described [5].

### Common variable immunodeficiency

CVID can be diagnosed in individuals from the age of 4 with hipogammaglobulinemia in at least two classes of Ig, always including IgG (<2 SD of normal values for age). Additionally, patients reveal poor production of specific antibodies and/or significantly reduced number of memory B cells after class switch recombination (<70% of the normal value for age). For definitive diagnosis, other causes of hypogammaglobulinemia and defects in cellular immunity should be excluded [2]. Infectious manifestations of CVID include recurrent respiratory tract infections, such as sinusitis, otitis, bronchitis, pneumonia, and gastrointestinal tract infections, i.e. *Salmonella*, *Giardia intestinalis*, and *Helicobacter* infections. Chronic bacterial bronchitis often cause bronchiectasis. Interstitial lung inflammation may lead to fibrosis of the lungs. Other infections occurring in CVID include meningitis, sepsis, osteomyelitis, urinary tract, joint, and skin infections [6]. What is more, non-infectious complications, including autoimmunity, interstitial lung disease, granulomatous disease, enteropathy, lymphoid hyperplasia, lymphoma, and/or cancer may be developed [7]. Autoimmune diseases occur in 40–60% of patients with a slight female predominance. The most common are cytopenias, including thrombocytopenia, anemia, and leucopoenia. In half of the cases, the hematological disorders, especially thrombocytopenia, may precede the diagnosis of CVID [8, 9]. Inflammatory bowel disease occurs in 30% of CVID individuals, and include gastritis, colitis, nodular hyperplasia, lymphoid infiltrates, and malignancy. Other common complications of CVID are non-infectious pulmonary lesions (interstitial lung disease, bronchiectasies, and pulmonary fibrosis), which are found in approximately half of the patients. Based on the histopathological picture of pulmonary lesions, the following can be distinguished: granulomatous lung disease, lymphocytic interstitial pneumonia, granulocytic-lymphocytic interstitial lung disease (GLILD) as a form of pulmonary lymphoid hyperplasia [10]. In 20% of patients with CVID, lymph node enlargement and splenomegaly are present. People with CVID have approximately a 1.8–5 times higher risk of developing lymphomas/cancers [11].

### Transient hypogammaglobulinemia of infancy

THI is defined as the decreased level of IgG with or without reduced IgA and IgM levels, occurring in children under 3 years of age. European Society for Immunodeficiency (ESID) requires THI patients to have reduced IgG levels, which recover around their fourth birthday [12]. Because of the requirement for spontaneous recovery, THI is a retrospective diagnosis. Children with this deficiency may not present any symptoms. THI is rarely associated with severe infections, but some individuals may suffer from recurrent sinopulmonary or gastrointestinal tract infections [13]. Most patients recover by age 2 years, but some may have decreased IgG levels until age 5 and occasionally even beyond that [14].

## Immunopathology and mechanisms leading to the disease

The precise patomechanism of CVID, sIgAD, and THI is still largely unknown. Since all of these disorders are associated with immunoglobulin production, a number of B-cell subset abnormalities have been shown, indicating B-cell differentiation disorders. Children with an early-onset of CVID showed a lack of an increase in the levels of memory B cells (CD19/CD27) and class-switched memory B cells (CD19/CD27/IgD/IgM) with age, as opposed to healthy children [15, 16]. Similar changes were shown in sIgAD children, yet only within the class-switched memory B-cell subsets [15, 17]. sIgAD patients showed also a decrease in the levels of terminally differentiated B-lymphocyte subsets [18]. These observations support the theory of a defect of class switching, terminal differentiation of IgA secretory plasma cells, and their long-term survival [17, 19]. THI children were shown to have normal B-cell subsets during the entire period of hypogammaglobulinemia [15, 20], yet others observed higher naive B-cell levels in THI patients [19].

**Figure 2.**
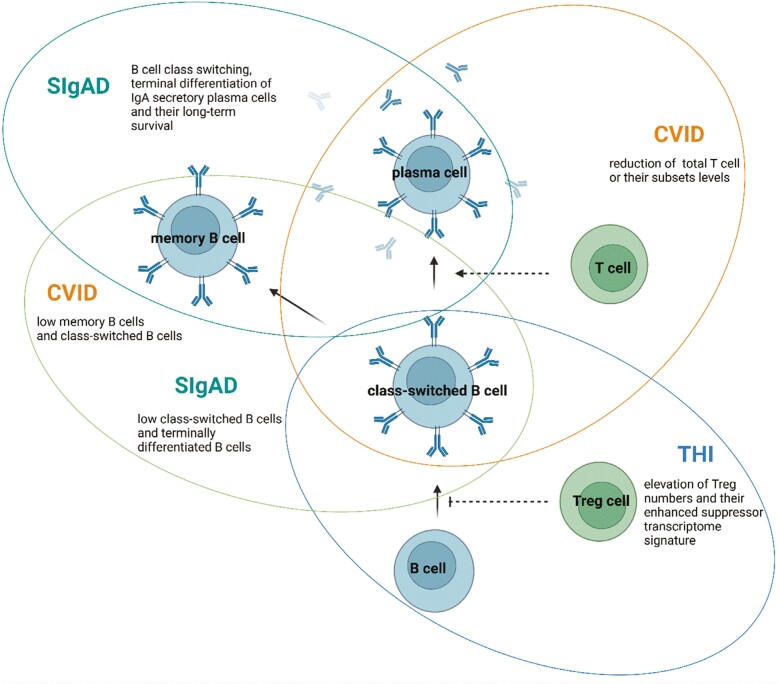
B- and T-cell cellular abnmormalities observed in CVID, sIgAD, and THI patients. Created with BioRender.com

Ig production disorder observed in CVID, sIgAD, and THI patients was also associated with the abnormalities in T-cell functions, as both Ig isotype switching and production can be influenced by different T-cell subsets [21]. As an example, Vlková et al. showed a positive correlation between an increase of some T-cell activation markers (HLA-DR, CD45RO) and a decrease in others (CD27, CD62L, and CD45RA), with a decreased number of memory and mature B cells in CVID patients [22]. Moreover, a reduction of total T cell or their subset levels in CVID patients was observed in a number of studies, and with a more profound deficiency in patients additionally exhibiting autoimmune disorders [23–25]. In the case of sIgAD, no differences in T cell and T-cell subsets levels comparing to healthy control subjects were reported [18, 26], including regulatory T (Treg) cell numbers [27]. The upregulation of Treg levels was, though, associated with the patomechanism of THI. Our group showed that Treg numbers in THI children are transiently elevated during the period of hypogammaglobulinemia, yet decrease with Ig levels normalization [27, 28]. The most recent transcriptome analysis of Treg cells isolated from THI patients indicated that these cells display enhanced suppressor transcriptome signatures, suggesting that THI pathomechanism might be associated with their enhanced regulatory and inhibitory functions [28]. B- and T-cell cellular abnmormalities observed in CVID, sIgAD, and THI patients are presented on Figure 2.

CVID is genetically heterogeneous, and only a small number of patients have an identified monogenic defect [29]. The *high*-*throughput* genomics studies highlighted the complex and often polygenic nature of this condition. More than 60 CVID and CVID-like genes have been identified over the last 20 years [30]. According to the latest IUIS classification, monogenic defects associated with the CVID phenotype are represented by 22 genes, some of them described in single patients (Table 1) [31]. The diversity of these genes reflects the complex requirements of B-cell biology as well as other immune compartments which may also influence B-cell homeostasis. Among them, there are genes encoding, i.e. key B-cell receptor costimulatory proteins (CD19, CD20, CD21, and CD81), tumor necrosis factor superfamily receptors, and ligands (i.e. TNFRSF13B/TACI, TNFRSF13C/BAFF-R, and TNFSF12/TWEAK), transcription factors mediating differentiation and cross talk (IKZF1, NFKB1, NFKB2), key regulators of actin dynamics (RAC2, ARHGEF1). Other genetic defects described as being associated with CVID show evidence of T-cell dysfunction, despite their predominant associations with antibody deficiency, such as cytotoxic T-lymphocyte antigen 4 (CTLA-4) and lipopolysaccharide (LPS)-responsive beige-like anchor protein (LRBA) deficiencies.

**Table 1.** Genes related to CVID phenotype according to IUIS 2023 [31] with associated clinical features and accessible treatment, based on series of case reports. Antibiotics, antifungal and antiviral drugs were not listed

CVID phenotype Disease	Genetic defect	Ig	Associated features	Accessible treatment
Activated p110 syndrome (APDS)	PIK3CD GOF	Normal/increased IgM, reduced IgG and IgA	Severe bacterial infections; reduced memory B cells and increased transitional B cells, EBV ± CMV viremia, lymphadenopathy/splenomegaly, autoimmunity, lymphoproliferation, lymphoma	IgRT -Rituximab -Tacrolimus -Rapamycin -Leniolisib -HSCT [32]
	PIK3R1		Severe bacterial infections, reduced memory B cells and increased transitional B cells, lymphadenopathy/splenomegaly, lymphoproliferation, lymphoma; developmental delay	
PTEN Deficiency (LOF)	PTEN	Normal/decreased	Recurrent infections, lymphoproliferation, autoimmunity; developmental delay	IgRT [33]
CD19 deficiency	CD19	Low IgG and IgA and/or IgM	Recurrent infections, may have glomerulonephritis (CD81 mutation abolishes expression of CD19, thereby phenocopying CD19 mutations)	IgRT [34, 35]
CD81 deficiency	CD81	Low IgG, low or normal IgA, and IgM		IgRT –Systemic steroids –Cyclophosphamide –MMF –Azathioprine [36]
CD20 deficiency	CD20	Low IgG, normal or elevated IgM, and IgA	Recurrent infections	IgRT (timely) [37]
CD21 deficiency	CD21	Low IgG, impaired anti-pneumococcal response	Recurrent infections	IgRT [38]
TACI deficiency	TNFRSF13B	Low IgG and IgA and/or IgM	Variable clinical expression and penetrance for monoallelic variants	IgRT [39, 40]
BAFF receptor deficiency	TNFRSF13C	Low IgG and IgM,	Variable clinical expression	IgRT/no IgRT [41]
TWEAK deficiency	TNFSF12	Low IgM and A, lack of anti-pneumococcal antibody	Pneumonia, bacterial infections, warts, thrombocytopenia. Neutropenia	NA [42]
TRNT1 deficiency	TRNT1	B cell deficiency and hypogammaglobulinemia	congenital sideroblastic anemia, deafness, developmental delay	NA [43]
NFKB1 deficiency	NFKB1	Normal or low IgG, IgA, IgM, low or normal B cells, low memory B cells	Recurrent sinopulmonary infections, COPD, EBV proliferation, autoimmune cytopenias, alopecia, and autoimmune thyroiditis	IgRT -Systemic steroids -MMF -Rituximab -CsA -Mesalazine -Azathioprine -Abatacept -HSCT [44]
NFKB2 deficiency	NFKB2	Low serum IgG, A and M; low B cell numbers	Recurrent sinopulmonary infections, alopecia and endocrinopathies	IgRT Systemic steroids MMF CsA [45]
IKAROS deficiency	IKZF1	Low IgG, IgA, IgM, low or normal B cells; B cells and Ig levels reduce with age	Decreased pro-B cells, recurrent sinopulmonary infections; increased risk of ALL, autoimmunity, CVID phenotype	IgRT [46] HSCT [47]
IRF2BP2 deficiency	IRF2BP2	Hypogammaglobulinemia, absent IgA	Recurrent infections, possible autoimmunity and inflammatory disease	IgRT Entocort [48]
ATP6AP1 deficiency	ATP6AP1	Variable immunoglobulin findings	Hepatopathy, leukopenia, low Copper	IgRT [49]
ARHGEF1 deficiency	ARHGEF1	Hypogammaglobulinemia; lack of antibody	Recurrent infections, bronchiectasis	IgRT [50]
SH3KBP1 (CIN85) deficiency	SH3KBP1	IgM, IgG deficiency; loss of antibody	Severe bacterial infections	NA [51]
SEC61A1 deficiency	SEC61A1	Hypogammaglobulinemia	Severe recurrent respiratory tract Infections	IgRT [52, 53]
RAC2 deficiency	RAC2	Low IgG, IgA, IgM, low or normal B cells; reduced Ab responses following vaccination	Recurrent sinopulmonary infections, selective IgA deficiency; poststreptococcal glomerulonephritis; urticaria	IgRT HSCT Lung transplantation [54]
Mannosyl-oligosaccharide glucosidase deficiency	MOGS	Low IgG, IgA, IgM, increased B cells; poor Ab responses following vaccination	Bacterial and viral infections; severe neurologic disease; also known as congenital disorder of glycosylation type IIb (CDG-IIb)	Supportive therapy [55]
PIK3CG deficiency (2 patients)	PIK3CG	Reduced memory B cells, hypogammaglobulinemia	Recurrent infections, cytopenia /lymphopenia, eosinophilia, splenomegaly, lymphadenopathy, HLH-like	Dexamethason, Anakinra [56]
BOB1 deficiency (1 patient)	POU2AF1	Reduced memory B cells, agammaglobulinemia with progressive tetraparesia	Recurrent respiratory infections, possible chronic viral infection of CNS	NA [57]
CTLA4 haploinsufficiency (ALPS-V)^#^	CTLA4	Decreased circulating B and T cells, Impaired function of Tregs	Autoimmune cytopenias, enteropathy, interstitial lung disease, extra-lymphoid lymphocytic infiltration, recurrent infections	IgRT Abatacept Rituximab Sirolimus Systemic steroids HSCT [58] Vedolizumab [59]
LRBA deficiency ^#^	LRBA	Reduced IgG and IgA in Most, Normal or decreased CD4 numbers T cell Dysregulation, Low or normal numbers of B cells	Recurrent infections, inflammatory bowel disease, autoimmunity	IgRT Abatacept Systemic steroids MMF CsA Sirolimus Azathioprine Adalimumab Sulfasalazine Hydroxychloroquine Splenectomy HSCT [60]

Abbrevations: IgRT, immunoglobulin replacement therapy; HSCT, hematopoetic stem cell transplantation; MMF, mofetil mycophenolate; CsA, cyclosporine A; NA, not available; #classified as regulatory T cell defects in diseases of immune dysregulation category.

The exact genetic cause of sIgAD and THI remains unknown and the pattern of inheritance is unclear, however, a family history of immunodeficiencies, especially for sIgAD or CVID in sIgAD patients, is a significant risk factor, as well as the evidence for a genetic predisposition [61, 62]. Associations between sIgAD and certain human leukocyte antigen (HLA) classes I, II, and III haplotypes have been described for many years [63]. However, it is currently believed that sIgAD is not associated with a distinct haplotype, rather the risk is conferred by the common extended HLA haplotype HLA-A1, B8, DR3, and DQ2 (the 8.1 haplotype) acting in a multiplicative manner [64]. The HLA-DR7, DQ2, and DR1, DQ5 haplotypes have also been shown to be the risk factors for sIgAD, whereas the DR15, DQ6 haplotypes have been reported to confer an almost complete protection against the disorder [65, 66]. Associations of non-HLA genes with sIgAD have been also demonstrated, although the particular variants of these genes were associated in parallel with autoimmune diseases like type 1 diabetes (T1D) and systemic lupus erythematosus (SLE)—Interferon Induced With Helicase C Domain 1 (IFIH1) or T1D and multiple sclerosis—C-Type Lectin Domain Containing 16A (CLEC16A). What is more, genome-wide association study meta-analysis identified new susceptibility loci for sIgAD like PVT1, ATG13-AMBRA1, AHI1, ICOS, and CTLA4 [67, 68] which may suggest that a complex network of genetic effects, including genes known to influence the biology of IgA production, contributes to sIgAD.

## Current treatment methods

CVID consists of two major clinical groups of patients: the first is characterized by high susceptibility to infections, while the second demonstrates no obvious susceptibility to infectious diseases but is characterized by non-infectious conditions including autoimmune, inflammatory and/or polyclonal lymphoproliferation, and increased risk of malignancy. Although infections in CVID can be reduced by immunoglobulin substitution with/without antibiotic prophylaxis, autoimmune diseases usually require additional treatment. Autoimmune cytopenias (autoimmune hemolytic anemia and/or thrombocytopenia, neutropenia, and pernicious anemia) are the most common autoimmune conditions observed in CVID patients. Autoimmune diseases are more likely in patients with CVID and monogenic defects: nuclear factor kappa B subunit 1 (NF-kB1), LRBA, CTLA4, phosphoinositide 3-kinase (PI3K), inducible T-cell costimulatory (ICOS), IKAROS, and interferon regulatory factor-2 binding protein 2 (IRF2BP2). IgG replacement therapy is the basic form of treatment for CVID, which reduces the mortality rate and the incidence of infections and bronchiectasis. The meta-analysis of Orange et al. showed that the incidence of pneumonia declined by 27% with each 100 mg/dL increment in trough serum IgG levels [69]. The initial dose of immunoglobulin is 0.4–0.5 g/kg body weight/4 weeks for intravenous (IV) form or 0.1 g/kg body weight/week for subcutaneous (SC) form. Facilitated delivery of an SC product by pre-treatment with hyaluronidase allows for larger doses to be delivered, usually every 3–4 weeks. However, both the intervals and dose of immunoglobulins require individual approaches to mainly protect from lower respiratory tract infections. Higher trough level (>7 g/L) and higher replacement doses may be needed for patients with complications such as enteropathy, bronchiectasis, and/or splenomegaly [69, 70]. Despite immunoglobulin supplementation, patients may suffer from infections, and in such cases introduction of antibiotic prophylaxis is necessary. Usually cotrimoxazol–trimetoprim or azithromycin are used. Apart from that in severe infections ciprofloxacin or inhaled tobramycin/colistin may be introduced [7]. The treatment strategies used for cytopenias in CVID are essentially the same as applied for immune-competent patients. First-line therapy generally includes intravenous steroids (1 g methylprednisolone) followed by moderate doses of oral steroids or high-dose IVIg (1–2 g/kg). A review by Cunningham–Rundles reported that most cases of immune thrombocytopenia (ITP) and autoimmune hemolytic anemia (AIHA) in CVID respond to oral or intravenous corticosteroids [71]. For patients with recurrent cytopenias, chronic treatment with intravenous or subcutaneous immunoglobulins reduces the probability of additional episodes; however, it was noted that a consistent IgG level of 7 g/L or more is required [72]. Another option for cytopenias includes thrombopoietin-receptor agonists (TPO-A), which have also shown benefit. It is suggested to use TPO-A as an alternative to splenectomy and rituximab in refractory cases of thrombocytopenia [73]. In many patients, ITP or AIHA is accompanied by an enlarged spleen or by cervical, mediastinal, or abdominal lymphadenopathy. Previously, it was believed that splenectomy in patients with CVID should be avoided due to a higher susceptibility to postoperative infections and sepsis with encapsulated microorganisms. Nevertheless, studies by Wong showed that if adequate immunoglobulin replacement is being continued, splenectomy has no association with adverse outcomes [74]. For refractory cases, some clinicians use immune-modulating treatment options such as growth inhibitors (azathioprine or mycophenolate mofetil). However, the risks of these drugs must be considered [74]. A much better option than the mentioned immunosuppressive drugs is provided by the monoclonal antibody B-cell depletion therapy (Rituximab). Although the risk of post-rituximab hypogammaglobulinemia and persistent B-cell lymphopenia is non-negligible, CVID patients are still on immunoglobulin supplementation, therefore this form of treatment may be preferable to others as the primary second-line therapy (after corticosteroids) [75]. There is one-case report of CVID patient who refractory to other treatment Evans syndrome was successfully treated with ustekinumab [76]. mTOR inhibitor, sirolimus, is recommended for the management of cytopenia in APDS and in other monogenic forms of CVID (LRBA deficiency and CTLA-4 haploinsufficiency), but also may be used in “typical” CVID [77]. Alternative therapy, based on CTLA-4-Fc fusion IgG1 immunoglobulin (abatacept) may be applied in patients with CTLA-4 and LRBA deficiency. The mechanism of this monoclonal antibody is based on the replacement of CTLA-4 and restoring the inhibitory function of T-cell activation [78]. The treatment of autoimmune neutropenia in CVID is not well established. High-dose IVIg, corticosteroids, or granulocyte-colony stimulating factor therapy can be used if neutropenia is severe (absolute neutrophil count < 500/mm^3^). Similarly to ITP, rituximab may also be used in therapy [9]. Another complication of CVID—granulomatous lymphocytic interstitial lung disease (GLILD) is concerned with inflammation and lymphoproliferation without any infective agent [79]. In most severe cases, it may lead to parenchymal lung damage [80]. The first step in therapy is to increase doses of immunoglobulin with higher trough levels (0.8–1 g/L). It may be helpful to prevent/reduce lung infections, but not interstitial lung disease. Systemic steroids can be introduced at the beginning of treatment but with limited benefit [81]. Therefore additional therapies are needed: e.g. rituximab, cyclosporine A, azathioprine, hydroxychloroquine, mycophenolate, infliximab, and abatacept. A combination of rituximab and mycophenolate or azathioprine has proven effective [82]. Apart from that, prophylaxis with macrolides may be beneficial [83]. There are also some data showing the good effect of sirolimus and abatacept in GLILD treatment [84, 85].

Apart from infections of the gastrointestinal tract (GIT), chronic bowel disease is the frequent complication in patients with CVID. Inflammation may be found in upper and lower GIT and is often difficult to control using standard IBD therapies. Lymphoid infiltrates, granulomas, nodular hyperplasia, IBD-like disease, villous atrophy may occur in approximately 32% of patients. Immunoglobulin replacement is not sufficient to control these complications. Low-dose corticosteroids or oral budesonide and immunosuppressants, including 5-aminosalicylate agents, 6-mercaptopurine (6-MP), or azathioprine (AZA), are usually used in inflammatory bowel disease. They are less effective for small bowel entheropathy [86]. Particularly in the case of granulomas anti-TNF agents (infliximab, adalimumab) may be useful [87, 88]. Although resemblance to gluten enteropathy has been noted in CVID enteropathy, gluten withdrawal is generally ineffective. There are also reports concerning the use of anti-integrin α4B7 monoclonal antibody (vedolizumab) in CVID steroid-refractory enteropathy [89]. Vedolizumab may improve gastrointestinal symptoms; however, it may exacerbate extra-intestinal inflammation [90]. IL-12 and IL-23 antagonists (ustekinumab) have been used with benefit in case of Crohn-like disease [91]. It is also very important to focus on adequate nutritional support, severe cases of malabsorption may require an elemental diet or total parenteral nutrition. There are some studies suggesting the role of gut microbiota in the etiology of CVID enteropathy [92]. However, the rifaximin treatment did not significantly influence on gut inflammation [93]. Unfortunately, for many patients with CVID enteropathy, the therapy is still unsatisfactory.

The most common cause of liver pathology in CVID is nodular regenerative hyperplasia leading very often to portal hypertension and cirrhosis. There is no other treatment than liver transplantation, but the outcome is not encouraging. Azzu et al. concluded that CVID patients undergoing liver transplantation had a higher mortality compared to the general population and a worse 5-year survival [94, 95].

Allogenic stem cell transplantation is the only form of therapy for CVID that guaranteeing a complete cure; however, its role in CVID treatment is still controversial. In the multicenter study conducted by Wehr et al. overall survival rate in CVID after HSCT was 48% after 2 years. Autoimmune conditions (i.e. autoimmune cytopenias and enteropathy), granulomatous disease, and hematological malignancies were the major indications to perform HSCT. The major causes of death were graft-versus host disease (GvHD), poor immune reconstitution, and infectious complications. On the other hand, IgRT was not necessary in 50% patients after HSCT, and the condition being the indication for HSCT resolved in 92% of surviving patients. This therapeutic approach could be beneficial in selected patients [96]. Froehlich et al. described a case of a female patient with CVID and autoimmune encephalitis, which was successfully treated by immunoablative conditioning and transplantation of autologous CD34-selected stem cells. There are no other data concerning autologous stem cell transplantation in CVID, but it seems that in patients with severe autoimmunity it could be the alternative form of treatment for those patients [97]. HSCT may be also a potential treatment for specific forms of CVID concerned with one gene mutations (i.e. LRBA).

There is no specific treatment for selective IgA deficiency and each patient should be managed individually and monitored for the development of CVID and/or autoimmunity. Intermittent or continuous prophylactic antibiotics may be helpful in patients with recurrent respiratory tract infections especially for patients with chronic sinusitis or bronchitis. IRT may be introduced if IgA deficiency is accompanied by IgG subclasses deficiency or impaired antibody responses to bacterial or vaccine antigens. Recommendations by the American Academy of Allergy, Asthma & Immunology (AAAAI) and the American College of Allergy, Asthma & Immunology (ACAAI) indicate necessity of allergy treatment in immunodeficiencies including sIgAD because allergic inflammation might facilitate the development of respiratory tract infections. Management of the allergies associated with sIgAD is similar to those in general. Apart from medications it is possible to use also immunotherapy and omalizumab [98]. Patients with sIgAD are more susceptible to autoimmune diseases (SLE, rheumatoid arthritis, thyroiditis, autoimmune hemolytic anemia, type 1 diabetes mellitus, Graves` disease, Crohn`s disease, ulcerative colitis, coeliac disease, autoimmune hepatitis, scleroderma, vitiligo, immune thrombocytopenic purpura, and autoimmune hemolytic anemia). Those diseases are treated as in other patients without IgA deficiency [99].

Despite expanding knowledge about possible pathomechanisms of sIgAD, there have been no particular advances or novelties in the therapy for several years and today there is no treatment for sIgAD but only for associated diseases.

THI may present with a broad spectrum of clinical manifestations, from the lack of symptoms to recurrent infections. Most patients recover spontaneously without any treatment by age 2 years but in some hypogammaglobulinemia persists until 5 years. Some children with THI finally turn out to have other deficiencies, e.g. IgG subclass deficiency, selective IgA deficiency, or selective antibody deficiency. Those patients may suffer from severe infections or autoimmune diseases. Hence, vigilance should be maintained [100, 101].

## The future of treatment

Identification of more genetic defects in patients with CVID in the future may give the opportunity to introduce specific targeted therapies especially for the management of various disease-related complications (Table 2). At present the wider use of sirolimus, JAK3-inhibitors (tofacitinib, ruxolitinib), monoclonal IL-12/23 antibody (ustekinumab), IL-23 antibody (guselkumab), may be discussed in all inflammatory complications in CVID patients [117]. Belatacept may be used in the same clinical conditions as Abatacept, in cases of excessive T-cell activation and inflammatory infiltrate formation [110]. Belimumab, an mAb that binds and neutralizes the B-cell survival factor BAFF, is approved for the treatment of SLE. In comparison to rituximab, it has a lower ability to deplete B cells. Perhaps it can be also used in CVID complications instead of rituximab [102]. Emapalumab is a fully human immunoglobulin G1 monoclonal antibody directed against interferon-gamma (IFN-gamma). It may be helpful in CVID complications concerned with T-cell dysregulation, but it increases the risk of infections. Apart from listened biologics, phosphodiesterase (PDE) inhibitors may have some clinical success in complicated CVID. Roflumilast is a selective PDE4 inhibitor indicated by the US Food and Drug Administration as a treatment in patients with severe COPD with chronic bronchitis and a history of exacerbations. Inhibition of PDE4 exerts an anti-inflammatory effect [118] and it also may be helpful in GLILD/LIP in CVID patients. It is possible that larazotide (AT 1001), which is investigated as a gut permeability regulator for the treatment of coeliac disease, would have a potential to alleviate symptoms of coeliac-like disease in CVID and coeliac disease in sIgAD [119].

**Table 2.** Biological agents which are used or may be potentially used in therapy of CVID complications

Name	Action	Complications of CVID	References
Rituximab	Anti-CD20	Granulomas, cytopenias, interstitial lung disease	[75]
Infliximab, Etanercept, Adalimumab	Anti-TNF	Granulomas, entheropathy, interstitial lung disease	[88] [102] [103] [104] [105] [106]
Bortezomib	Proteasome inhibitor	Cytopenias, lymphoproliferative disorder	[76] [107]
Vedolizumab	Anti-integrinα4β7	Entheropathy	[89] [90] [108]
Ustekinumab	Anti-IL-12/23	Enteropathy	[91]
Abatacept	CTLA-4 FcIgG1	Interstitial lung disease, LRBA, and CTLA4 deficiency	[84]
Belimumab	Anti-BAFF	NR, SLE	[102]
Guselkumab	Anti-IL-23	NR, psoriasis, psoriatic arthritis	[109]
Belatacept	CTLA-4 FcIgG1	NR, kidney transplantation	[110]
Emapalumab	Anti-INF-γ	NR, primary hemophagocytic lymphohistiocytosis	[111]
Tofacitinib	JAK-inhibitor	NR, rheumatoid arthritis, psoriatic arthritis, ankylosing spondylitis, ulcerative colitis,	[112] [113] [114]
Ruxolitinib	JAK-inhibitor	NR, myelofibrosis	[115]
Leniolisib	PI-3K inhibitor	activated PI3Kδ syndrome (APDS)	[116]

Abbreviations: NR, not reported in CVID treatment.

As regards sIgAD and THI, there have been no reports of new therapies in recent years, but a better understanding of molecular mechanisms of these deficiencies perhaps will bring some new advances.

## Conclusion/discussion

Although we discussed three immunodeficiencies: CVID, sIgAD, and THI, the currently used therapy and therapeutic future perspective is mainly focused on CVID and the treatment of its complications. It is because sIgAD and THI do not cause such serious, sometimes life-threatening symptoms, as CVID does. The diversity of possible therapeutic models results from a great heterogeneity of the disease variants, implying the need for personalized medicine approach as a future of CVID treatment. When undertaking therapy, the clinicians often rely on case reports, whereas randomized control trials are usually very scarce. Thus, the close collaboration between different research groups dispersed in different countries is highly desired.

Currently, the most urgent need is the optimization of treatment of granulomatous disease, a major challenge among complications of CVID. There is also a constant necessity of development of new antibacterial drugs because of the enhancing resistance by the microbes. The microbial resistance will become a real challenge, especially in immunodeficiencies where antibiotics are used frequently and in a prolonged manner. Prevention of COVID-19 recurrence in CVID patients is also a challenge.

## Data Availability

There are no new data associated with this article.

## Abbreviations

AIHA: autoimmune hemolytic anemia
CVID: common variable immunodeficiency
GLILD: granulocytic-lymphocytic interstitial lung disease
GvHD: graft-versus host disease
GWAS: genome-wide association study
HLA: human leukocyte antigen
IFIH1: interferon induced with helicase C domain
ITP: immune thrombocytopenia
LIP: lymphocytic interstitial pneumonia
NRH: nodular regenerative hyperplasia
sIgAD: selective IgA deficiency
SLE: systemic lupus erythematosus
T1D: like type 1 diabetes
THI: transient hypogammaglobulinemia of infancy
UCH: unclassified hypogammaglobulinemia
